# Foreign Correspondence

**Published:** 1857-04

**Authors:** W. F. Westmoreland

**Affiliations:** Professor of the Principles and Practice of Surgery in the Atlanta Medical College


ATLANTA
### Medical and Surgical Journal.

Vol. II.] APRIL, 1857. [No. 8

### ORIGINAL COMMUNICATIONS.

### ARTICLE I.

#### FOREIGN CORRESPONDENCE.

By W. F. Westmoreland, M. D., Professor of the Principles and Practice of Surgery in the Atlanta Medical College.

Paris, January 10th, 1857.

*Dear Doctor*—Much has been said, and justly so, in praise of the arrangement of the hospitals of Paris. As exhibiting the progress of science, and affording advantages to the medical student, they are, perhaps, the most perfect of any in Europe; the attending physician and student having many important privileges ceded them here, that are denied them in other cities; still, they are, in some particulars, defective. One defect, and the only one that I shall notice here, is the absence of any special wards or hospital for the treatment of diseases of the eye; patients laboring under this important class of disease being distributed indiscriminately in the various surgical wards. For almost every other important class of disease, there are certain wards; and for some, certain hospitals are set apart for their treatment; such wards being under the control of physycians or surgeons devoting themselves entirely to certain classes of affections, each receiving only such cases as come directly under their speciality, as the venerial hospital, the scrofulous department at the Hopital des Enfants Malides; diseases of the skin at St. Louis; wards for disease of the bladder and urethra, under the care of M. Civiale, at Neker, &c., &c. Why it is that this defect, preventing the connection of oculists with the hospitals, and thus throwing them upon their own resources for improvement, has been so long overlooked, I am not able to say; one thing, however, is certain, that it cannot be for the want of importance of this class of disease.

This defect, so far as regards the medical student, is, to a great extent, obviated by the establishment in the city of several eye clinics upon private resources, at which is congregated three or four times a week a number of patients, the student having, by paying a few francs per month, every facility of studying the diseases of this important organ. It is of these clinics, or rather that of M. Desmorres, that I wish more particularly to speak in this letter.

M. Desmorres is certainly one of the first oculists in France, and has done as much for this particular department of science as any surgeon of the present day. His clinic, to which I have been attached ever since my arrival in Paris, is, without doubt, the best in the city, he receiving more patients, perhaps, than all the others combined—the number examined and prescribed for upon the days of the clinic, frequently amounting to between two and three hundred, a sufficient number, certainly, to experiment upon a large scale.

For the past several years, M. Desmorres has been devoting considerable attention to the treatment of lachrymal fistula, has experimented extensively, with a view of determining the value of the various plans of treatment proposed for the cure of this rebellious affection. The result of these experiments, and they have been upon an extensive scale, has been such as to condemn the, at present, most popular method, which consists in dilating the lachrymal duct by means of a stylet. He remarked a few days ago, in a lecture—and since in a private interview, told me the same--that of the hundreds that he had treated by this method, he knew of but *one* case where the cure had been radical. He said that it was possible that there were other cases that he had lost sight of, but that if any existed, they must certainly be very rare, as it was his misfortune to see them return at from a few months to a year after the treatment was discontinued. He has varied the plan of dilating the duct—has used stylets of various forms and substances, continuing the treatment from eight months to a year, and always with the same unfavorable result.

He contends that those who have had such happy results from this plan of treatment, have mistaken a temporary relief for a radical cure—a relief which, as a general rule, does not last so long as the treatment of which it is the result—the duct, in a few months after the treatment is discontinued, presenting the same obstruction as before its commencement. He renounces, then, this plan of treatment as a means of radical cure, resorting to it when, from some circumstance connected with the patient, it is thought best to attempt only temporary relief.

The method of permanent dilatation, by means of the canula of Dupuytren, he considers also objectionable from the numerous inconveniences with which it is attended, there arising, sooner or later, accidents which make it necessary to extract the canula. In some cases, the canula, a day or two after the operation, is displaced, intervening with the cicatrization of the incision for its introduction, in a few cases, this tendency persisting to such an extent as to prevent the success of the operation. In other cases, the canula descends, passing through the canal, and falling into the nasal fossa, the pharynx or the larynx; or, as in a case operated on by M. Velpeau, passing through the palatine processes of the superior maxillary bone, and falling into the mouth. Abscesses at the internal angle of the eye, calling for the prompt removal of the canula, are not unfrequent.

The above are some of the accidents which may arise at from a few days, to a few weeks after the introduction of the tube. Later, the canula may become obstructed by a calcareous deposit, thus exciting an inflammation of the parts, which continues until the foreign body is removed, or as is more frequent, the tube is obstructed by a sort of hypertroply of the mucous membrane of the sac and duct, which covers entirely the superior extremity of the canula, and thus preventing the passage of the lachrymal fluid. But few surgeons of the present day are partisans of this method of operating.

Several other operations have been proposed and practiced, as the perforation of the os unguis, by Woolhouse, the process of M. Langier, in which he penetrates the maxillary sinus with a trocar, the tear reaching the nasal fossa by dropping through this sinus; but, as it is not my purpose here to discuss the various plans of treatment, but rather to give that usually resorted to by M. Desmorres, I shall let them pass for the present. M. Desmorres' favorite method consists in the complete obliteration of the lachrymal sac, either with the chloride of zinc or the actual cautery.

But, if the lachrymal sac is destroyed, what will be demanded by the adversaries of this plan of treatment? what will become of the tears? Or, as I was asked by a distinguished surgeon of the United States, when speaking of this operation, if I regarded the lachrymal sac and duct of so little importance, as to consider it rational to council their destruction? In answer to the latter, it might be asked, for example, if any one doubts the importance of one of the inferior extremities; and again, if any one, for a moment, doubts the propriety, under quite a variety of circumstances, of sacrificing this important member? The sac, as the leg, when sacrificed is diseased, and to such an extent as to interfere with its proper functions—the surgeon, in either case, interfering with the hope of placing the patient in a more comfortable position. In answer to the first, as to what is to become of the tears, it is necessary, first, to determine their origin; secondly, to know if they are secreted continuously; and thirdly, what is the part the lachrymal duct performs in their excretion. To discuss, as I would like, these physiological points, would require more time and space than I can possibly devote to the subject; I shall then content myself for the present by giving, in a very few words, the views of M. Desmorres, with some of the facts in support of his views.

He contends that the lachrymal gland is not the only source of the tears—that the experiments of Magendie and Martini, upon animals, in which they extirpated the lachrymal gland, are in support of this opinion; they find the eye, in each case, equally well lubricated after, as before the extirpation—the secretion of the tears, to all appearance, being equally abundant. The extirpation of the lachrymal gland in the human subject has, so far, given the same result. In this extirpation by Harpin, Daviel, O'Beirne, Lawrence, de Graefe, Bernard, Laugenbeck, and others, no case has been observed in which the eye presented the least dryness. In the case reported by Harpin, if the conjunctiva, after the extirpation, was touched with the end of a probe moistened with the tincture of opium, the eye would instantly fill with tears, and to such an extent as to overflow. It is evident then, from the above facts, that the lachrymal gland plays only a secondary part in the secretion of the tears; the greater part being secreted by the vessels of the conjunctiva. As an evidence of their secretion by the conjunctiva, if we touch that membrane so as to remove the fluid which protects it, we will instantly see appear upon its surface, thus touched, small drops of liquid, which is readily recognized as the tears.

Another evidence, if any other is wanting, in support of the part the conjunctiva has in the production of the tears, is, that in the affections of the gland, and as we have seen, even in its complete extirpation, there never results *xérosis*, a dryness so dangerous to this organ, and which frequently has place when, from a continuous inflammation, from whatever cause, the conjunctiva presents an extensive cicatrix.

Does the lachrymal gland secrete constantly? M. Desmorres contends that the structure of this gland is the same as the salivary glands, and, like them, secretes periodically, that is, when the surfaces upon which they pour their contents are irritated; regarding the lachrymal gland as a sort of reserve, destined to furnish a flow of liquid sufficient when the conjunctiva is irritated, to rid it of any foreign body that might have lodged upon its surface. That, ordinarily, when there is no irritation about the eye, the gland rests inert, leaving the conjunctiva to furnish alone the necessary amount of fluid to lubricate the eye. M. Hyrtl, of Vienna, advances the same views in regard to the functions of this gland.

It rests alone, now, of the three propositions, to determine the part the sac and duct has in the excretion of the tears. Under ordinary circumstances—that is, when there is no irritation about the eye—the mucous membrane being lubricated by secretions from its own surface, the fluid thus secreted passes off by evaporation. MM. Desmorres and Hyrtl have observed, that, in this normal condition of the parts, there is no fluid to be found, either in the puncta lachrymalia or in the lachrymal canals. The strongest evidence, however, of the evaporation, under ordinary circumstances, of the liquid lubricating the conjunctiva, is the complete absence of epiphora, under the same circumstances, after the complete obliteration of the lachrymal sac. It is then only during a hypersecretion of the tears, from various causes, as a bleak wind, dust, foreign bodies, and many other circumstances which tend to irritate and inflame the conjunctiva or other tissues of the eye, that the lachrymal canals serve to conduct the lachrymal fluid. One of the most prominent symptoms of an obstruction of the lachrymal duct is epiphora. This would appear, without reflection, to be in opposition to the above deductions, and is frequently urged by those opposed to the destruction of the lachrymal sac. As we have seen above, and as all admit, the least irritation about the eye induces a hypersecretion of the lachrymal fluid—this secretion being in proportion to the amount of irritation. In an obstruction of the lachrymal duct, we have all the conditions necessary to induce this hypersecretion; as from the first distention of the sac there is more or less irritation which increases with the obstruction, and, finally, in quite a number of cases, resulting in an abscess of the sac. Epiphora, then, so constant in this lesion, is not so much from the obstruction preventing the passage of the tears, as their increased secretion induced by the irritation of the parts. All know how frequent it is to see the eye overflow with tears from the least irritation, as a bleak wind, dust, &c., even when the lachrymal apparatus is in a normal condition. The proposition for which M. Desmorres contends, is the removal, definitively, of the irritation and inflammation of the sac by its complete obliteration. He has, within the past ten years, adopted this method of treating lachrymal fistula in quite a number of cases, and has demonstrated that the increased flow of tears ceases with the removal of the irritation, the patient being cured with the inconvenience of a slight overflow of tears when, from any circumstance, the conjunctiva is excited.

This method is not original with M. Desmorres; it was proposed and practiced by Angelo Nannoni, of Florence, as far back as the middle of the last century. The ancients, too, treated lachrymal fistula as they did any other fistulous opening by the cautery, without a correct knowledge, however, of the lachrymal apparatus.

In the obliteration of the sac by the actual cautery, the sac is freely opened by an incision near an inch in length, commencing a few lines above the tendon of the orbicularis, freely dividing the tendon so as to reach the superior portion of the sac; the incision should be, to some extent, curved, the concavity looking towards the eye. After the hemorrhage is arrested, the incision is dilated by means of two hooks, the eye protected by a compress or piece of paste-board, and the cautery freely applied to the whole extent of the sac. Ice or cold water, by means of a bit of cloth or lint, should be constantly applied for the first twenty-four hours after the operation.

In the obliteration of the sac, by means of caustics, of which M. Desmorres gives preference to the chloride of zinc, the incision need not be so extensive, and may be commenced immediately below the tendon of the orbicularis. The caustic, if the chloride of zinc be used, may be introduced by means of a goose quill or any other tube, care being taken that the caustic does not come in contact with the edges of the incision. The same precautions, to prevent a too violent grade of inflammation, should be observed, as we have above suggested in the process by the actual cautery. M. Desmorres insists upon the importance of the obliteration of the superior portion of the sac, for reasons that are readily understood.

Much might be said in regard to the particular indications, giving preference to one or the other methods of obliterating the sac, but as I have already extended this letter much longer than I expected, I will, for the present, close.

---

PARIS, JANUARY 20TH, 1857.

In my last letter, I proposed to give you the treatment of some of the more important diseases of the eye, as practiced at the clinic of M. Desmorres; but, if I am not mistaken, I, in that letter, discussed, to the exclusion of everything else, obstructions of the lachrymal duct. I shall, then, in this, continue my notice of this clinic, commencing with that very common, but important, lesion, cataract.

All have long since renounced the possibility of removing the opacity of the crystalline lens by any medication, either local or general, agreeing that, sooner or later, an operation will be demanded; but, when we come to discuss the manner of operating, we do not find such harmony—every surgeon having his favorite method, which he strenuously contends has its advantages. Of the various operations proposed, we may make two great divisions: one including the extraction of the lens by whatever procedure; the other embracing the various operations in which the lens is left either in the posterior or the anterior chamber. A review of the various methods embraced in these two grand divisions would not be without interest, but as it is my object here to speak alone of those practiced at the clinic of M. Desmorres, I shall proceed at once to their discussion.

M. Desmorres is a strenuous advocate of the extraction of the lens, adopting this method under almost all circumstances. The operation by extraction has been usually restricted to uncomplicated cases of hard cataract. M. Desmorres goes for the extraction of soft cataract as well as that variety of hard cataract in which there are adhesions between the capsule and the iris. His operations by extraction in soft cataract, so far as I have seen, have been more satisfactory than in the most favorable cases of hard cataract. He has three distinct operations for the extraction of the lens: first, the operation for the extraction of uncomplicated hard cataract, which is the usual operation for extraction; secondly, an operation for the extraction of soft cataract; and, thirdly, a method differing from the other two, which he practices for the extraction of hard cataract, complicated with adhesions between the capsule of the lens and the iris, an accident so frequent in chronic iritis.

The first, or the operation which he proposes in cases to which the method by extraction has been usually restricted, differs from the ordinary method for extraction only in the *manuel opératoire.* Thus, instead of completing the section of the cornea with the cataract knife, as is usual, he withdraws the knife before the section is complete—leaving a small point at the superior portion of the flap still in tact. After waiting a few minutes, that the muscles of the globe of the eye may cease to contract with such force, he completes the section with a very delicate probe pointed knife. He, as the majority of surgeons who perform this operation, insists upon the advantages of the superior over the inferior section—contending that in the inferior section the incision in the cornea is constantly bathed in the secretions of the eye, which necessarily accumulate at this point, interfering with the process of adhesion, and in a number of cases producing a separation of the wound. Again, in the inferior section, the least motion of the lids tend to displace the flap, while in the superior section, if the eye is closed, the superior lid tends to keep the flap in place. In two cases I have seen M. Desmorres, in this operation, extend the flap upon the conjunctiva, extracting the lens without completing the section.

His operation for the extraction of soft cataract, which he terms "*extraction linéaire*," consists in a section of the cornea near the sclerotic with a lancet-shaped knife, as in the operation for artificial pupil; after the section, which is a straight incision, consequently the name *lineaire*, a delicate hook is introduced into the anterior chamber, and the anterior surface of the capsule ruptured in several places. If a small curette is now introduced into the incision, or is passed against the external lip, so as to separate its edges, at the same time that slight pressure is made upon the globe of the eye, the lens, if sufficiently soft, instantly escapes through the incision. If the lens be less soft, the consistence of jelly, for example, it may be necessary to introduce the curette for its extraction. M. Desmorres insists upon the importance of not rupturing the posterior surface of the capsule, contending that if there is the least wound of the posterior surface of the capsule, the vitreous humor instantly projects, displacing the softer lens behind the iris, and thus preventing the possibility of its extraction. The most beautiful results that I have ever witnessed from any operation for cataract, have been from this method. I have seen patients leave the clinic at from twenty-four to forty-eight hours after the operation, with complete union of the cornea, they able to see their way.

When there are, from any cause, firm adhesions between the capsule and iris, the pupil permanently fixed, it is evident that the lens cannot be extracted in the usual way; neither can an operation with the needles be performed with any prospect of success. In such cases, M. Desmorres proposes a double operation—artificial pupil with extraction.

His usual plan of performing this operation is to proceed as in artificial pupil, in fact, performing first the operation for artificial pupil by *déchinement*, making the incision in the cornea, perhaps, more extensive than is usual in the operation, and after, to extract the opaque lens through the opening thus made in the iris. The operation is performed as follows: With a lancet-shaped knife, make an incision in the cornea as near the insertion of this membrane into the sclerotic as is convenient; the section made, with a delicate pair of curved forceps, introduced through the incision, as large a portion of the iris as is possible, is seized near the pupil, and, by a quick movement, is brought out through the incision in the cornea and clipped off with a pair of scissors. The iris in this operation is torn, the rupture of the membrane following the direction of the fibres converging towards the pupil, thus leaving a triangular opening, the apex of which is directed towards the pupil. Through the opening thus made in the cornea and iris, a small curette is introduced behind the lens, and after breaking up the remaining adhesions, the lens, before its extraction, is broken into several pieces, if possible, by pressing it against the cornea.

I have seen this operation performed in two cases, and in both with success.

I should have mentioned, when speaking of the usual operation for hard cataract, that M. Desmorres never dilates the pupil in the operation, concluding that the lens is readily extracted without its dilatation, and with less danger of the escape of the vitreous humor. Neither does he *prepare* the patient, as its termed, for any operation, by a system of dieting, rest, purgation, &c., as is so frequently practiced by surgeons. Says that the best time to operate is when the patient is in the most perfect state of health—that by interfering with the general habits of the patient by diet and repose, or by the administration of a purgative, we derange, to a greater or less extent, the general health, and, in a number of cases, favor the development of accidents which we attempt to prevent. I have seen him several times perform the operation for cataract by extraction, upon patients who had but an hour or two before left their usual occupation, and without accident.

Since the discovery of the *opthalmoscope*, by Halmholtz, in 1851, M. Desmorres has been constantly engaged in the study of the before obscure diseases of the internal eye by means of this instrument. By constant application and improvements in the opthalmoscope, he has arrived at such perfection, that he examines with the same facility, and makes his diagnosis with the same precision of diseases of the internal eye, as the choroid membrane, retina, *papilla* of the optic nerve, &c., &c., as the disease of the external membranes. To present the many advantages that has resulted from this discovery would require many pages. It has done as much and perhaps will do more for diseases of the internal eye, than the great discovery of Laennec and Avenbrugger has done for diseases of the chest. M. Desmorres strikes from his nosology that elastic word, amaurosis, which has for centuries served to cover the ignorance of oculists of the many lesions of which it is only a symptom.

